# Anoxygenic Photosynthesis —A Photochemical Reaction That Does Not Contribute to Oxygen Reproduction—

**DOI:** 10.1264/jsme2.ME3101rh

**Published:** 2016-03-03

**Authors:** Satoshi Hanada

**Affiliations:** 1 Graduate School of Science and Engineering, Tokyo Metropolitan University1–1 Minami-Osawa, Hachioji, Tokyo 192–0397Japan

Plants, algae, and cyanobacteria perform photosynthesis with the resultant production of oxygen, which supports all organisms that consume it through their respiration. This type of photosynthesis is an indispensable part of the global oxygen flux. However, another type of photochemical reaction, photosynthesis without oxygen production, exists, and has been designated anoxygenic photosynthesis. A number of ecologists regard anoxygenic photosynthesis as a negligible photochemical reaction because it contributes nothing to the reproduction of oxygen. This type of photosynthesis does not appear to have any beneficial function.

The global impact of anoxygenic photosynthesis is considered to be negligible. Nevertheless, organisms in the domain *Bacteria* perform this type of photosynthesis, with some dilettantes referring to them as anoxygenic phototrophic bacteria. Contrary to all expectations, studies on anoxygenic phototrophic bacteria have a long history in microbiology. Anoxygenic phototrophic bacteria were discovered at the dawn of microbiology more than 100 years ago. The first anoxygenic phototrophic bacterium was identified in 1901 and was subsequently described as *Rhodospirillum* species by the German botanist, Hans Molisch in 1907 (10). *Rhodospirillum* species are purple bacteria that belong to the phylum *Proteobacteria*, and the term “purple bacteria”, which refers to a major group among anoxygenic phototrophic bacteria, was also simultaneously proposed by Molisch. Another major anoxygenic phototrophic group, green sulfur bacteria, which belong to the phylum *Chlorobi*, was described by the Russian biologist, Georgii A. Nadson in 1906 (13). Martinus W. Beijerinck, a famous microbiologist in the Netherlands, who was a contemporary of Molisch and Nadson, proposed a lactic acid bacterial group, the genus *Lactobacillus*, in 1901.

None of the anoxygenic phototrophic bacteria have the ability to use water as an electron donor (or cannot oxidize water), and, thus, perform photosynthesis using sulfide, hydrogen or organic substrates. Therefore, photosynthesis by these bacteria does not involve oxygen. In the domain Bacteria, oxygenic photosynthesis is limited to only one phylum, *i.e.*, *Cyanobacteria*. On the other hand, anoxygenic photosynthesis is widely distributed over several bacterial phyla. Purple bacteria in the phylum *Proteobacteria* and green sulfur bacteria in the phylum *Chlorobi* described above inhabit various environments such as soil, ocean, lakes, and rivers. Some species have even been discovered in hot springs (7, 25), and a culture-independent survey revealed that a brackish lake was abundant in a number of green sulfur bacteria (12). In addition to these bacteria, there are two groups, i.e., thermophilic or mesophilic phototrophs showing filamentous morphologies, designated as filamentous anoxygenic phototrophs belonging to the phylum *Chloroflexi* (4), and spore-forming anoxygenic phototrophs called heliobacteria are included in the phylum *Firmicutes* (3). Anoxygenic phototrophs have recently been discovered in two phyla *Acidobacteria* and *Gemmatimonadetes*, *i.e.*, *Chloracidobacterium thermophilum* isolated from a hot spring (23) and *Gemmatimonas phototrophica* from a lake (28), respectively. Therefore, the phylogenetic extent of anoxygenic phototrophic bacteria is larger than expected.

Anoxygenic phototrophic bacteria vary broadly not only in terms of their phylogenetic positions, but also in the compositions of their photosynthetic apparatuses. Organisms performing oxygenic photosynthesis, *i.e.*, cyanobacteria, algae, and plants, basically share a common mechanism for photosynthesis; it is performed in algae and plant chloroplasts, which originated from an ancestor of cyanobacteria. They have chlorophyll *a* as the essential photo-pigment and two types of photochemical reaction centers called photosystems I and II. However, anoxygenic phototrophic bacteria possess bacteriochlorophyll(s) instead of chlorophyll and contain one of two photosystems because anoxygenic photosynthesis only requires one type of photochemical reaction center. Phototrophs belonging to the phyla *Chlorobi* (green sulfur bacteria), *Firmicutes* (*Heliobacterium* spp.) and *Acidobacteria* (*C. thermophilum*) contain photosystem I only (23). On the other hand, those in the phyla *Proteobacteria* (purple phototrophic bacteria), *Chloroflexi* (filamentous anoxygenic phototrophic bacteria), and *Gemmatimonadetes* ( *G. phototrophica*) have photosystem II (28).

In addition to photochemical reaction centers, clear differences have also been reported in the light-harvesting systems that define the absorption band of light. Almost all phototrophic members in the phyla *Chlorobi*, *Chloroflexi* and *Acidobacteria* possess a special light-harvesting unit called a chlorosome and mainly use infrared light at 740–750 nm. Heliobacteria in the phylum *Firmicutes* absorb slightly longer wavelengths (at 786–792 nm) than those of the chlorosome-equipped phototrophs. Purple phototrophic bacteria and *G. phototrophica* use the longest region of infrared light for photosynthesis (at 800–1020 nm). Therefore, different types of anoxygenic phototrophic bacteria may co-exist in the same environment because they share light based on to their own absorption bands. The structural and pigmentary diversities found among anoxygenic phototrophic bacteria have been attributed to their long evolutionary history. Anoxygenic photosynthesis appeared prior to the emergence of oxygenic photosynthesis, and has evolved over an extremely long period of time.

Some phototrophic organisms are considered to be more useless than anoxygenic photosynthetic bacteria, and have been designated as aerobic anoxygenic phototrophic bacteria (AAnP bacteria). AAnP bacteria possess a photosynthetic apparatus, but are unable to support their growth by photosynthesis only. They are strictly aerobic heterotrophs that grow via respiration, notwithstanding their ability to photosynthesize. The first AAnP bacterium, *Erythrobacter longus*, was discovered by Shiba and Shimidu in 1982 (20). This discovery was followed by the proposal of two species belonging to the same genus, *i.e.*, *Roseobacter litoralis* and *R. denitrificans* in 1991 (21). Although these three species were detected in marine environments, related AAnP bacteria have also been isolated from freshwater environments such as *Porphyrobacter neustonensis* (2) and *Erythromicrobium ramosum* (27).

These AAnP bacteria cannot produce oxygen, and are also unable to fix carbon dioxide. They make no contribution to oxygen recycling or carbon dioxide reduction, and appear to be negligible phototrophs in the global material and energy flux. However, these phototrophs vigorously thrive in aquatic environments. Several reports based on culture-dependent and -independent studies have suggested that a large number of AAnP bacteria inhabit oligotrophic marine (19) and freshwater (6) environments. Although the reasons why AAnP bacteria significantly dominate in these environments currently remain unclear, a proteomic analysis of *R. litralis* suggested that it possesses the capacity to regulate its metabolism in accordance with illumination (29), and the viability of an AAnP bacterium in the light was reportedly higher than that in the dark under nutrient-deficient conditions (22). The photosynthetic apparatus that they use appears to generate energy in order to survive starvation when light is provided. These features may be advantageous to AAnP bacteria and, thus, they may outcompete other non-phototrophs in oligotrophic environments. A similar increase in viability by illumination has been reported in anoxygenic phototrophic bacteria such as *Rhodopseudomonas palustris* (5, 8). AAnP bacteria that do not produce oxygen or fix carbon dioxide do not appear to contribute to the global ecosystem. However, they are still a considerable bacterial group due to their carbon flux in oligotrophic environments.

These AAnP bacteria have been found in authentic genera recognized as non-phototrophic groups. The genus *Bradyrhizobium*, which consists of root and stem-nodulating bacteria, for example, contains strains exhibiting aerobic anoxygenic phototrophy, *e.g.*, strains BTAi1 (1), ORS285 (11) and S23321 (15), as well as non-phototrophic, nitrogen-fixing species (17, 24). This type of phototrophy has also been observed in the genus *Methylobacterium*, which comprises methylotrophic aerobes. *Methylobacterium radiotolerans* expresses a photosynthetic apparatus (14, 18), and genome analyses have revealed that several species in this genus including *M. extorquens* possess a complete gene set for anoxygenic photosynthesis (9, 16). Although these bacteria lack the ability to grow by photosynthesis only, similar to other AAnP bacteria, strains belonging to the genus *Bradyrhizobium* have the ability to fix atmospheric nitrogen and supply plants with ammonium for their synthesis of amino acids. Furthermore, species in the genus *Methylobacterium* also make organic matter from simple compounds, *e.g.*, methanol, by means of unique methylotrophic metabolic pathways, and promote plant growth (9).

The fixation of nitrogen by anoxygenic phototrophic bacteria has been reported, and some species living in the soil have been shown to contribute to the growth of plants under low fertile conditions (26). In addition, a large number of anoxygenic phototrophic bacteria have the ability to fix carbon dioxide and produce organic substrates. Anoxygenic photosynthesis is an essential part of the terrestrial ecosystem and plays an important role in the global flux of carbon, nitrogen, and possibly sulfur, but never produces oxygen. These bacteria absorb extraterrestrial light energy from the sun, convert it into biochemical energy, and adapt to the terrestrial ecosystem. A large number of anoxygenic phototrophic bacteria (including AAnP bacteria) thrive in all environments worldwide; however, the reason why they are dominant in some oligotrophic environments remains unclear and their ecological roles have not yet been determined. Moreover, the huge diversity that exists in the function and pigment of anoxygenic phototrophic bacteria represents an interesting research target to provide an insight into the early evolution of photosynthesis. Anoxygenic photosynthesis does not contribute to global oxygen reproduction, but is still a biological component that is indispensable for the terrestrial ecosystem as well as the recycling of essential substrates other than oxygen.
